# A Case of Lower Extremity Weakness with Foot Drop Following Intramedullary Nailing of a Pathologic Femoral Fracture

**DOI:** 10.7759/cureus.6561

**Published:** 2020-01-04

**Authors:** Charles A Gusho

**Affiliations:** 1 Orthopaedic Surgery, Medical College of Wisconsin, Green Bay, USA

**Keywords:** intramedullary nailing, metastasis, pathologic fracture

## Abstract

A 62-year-old woman presented with a pathologic femoral fracture. She underwent bilateral femoral intramedullary nailing (IMN) fixation, and her postoperative course was complicated by worsening lower extremity weakness and foot drop. Lumbar imaging identified vertebral compression fractures with foraminal encroachment. The patient was thereafter started on a radiotherapy regimen and discharged home. Pathologic fractures most commonly occur in the proximal femur. In some patients, contralateral weakness suggests an additional fracture, though it may represent spinal involvement. This case explores multiple current treatment options for pathologic fractures of the femur and spine and suggests that prophylactic endoprostheses may have greater selective benefit than IMN fixation.

## Introduction

The incidence of metastatic disease will likely increase in the next 20 years with the aging population. Bony metastasis most commonly occurs in the proximal third of the femur, resulting in a pathologic or impending fracture [1]. When one presents with a pathologic femoral fracture, however, it is important to discern lower extremity weakness from a spinal etiology, as this may differentially affect the surgical plan and necessitate a more expeditious intervention. Presented here is a case of bilateral lower extremity weakness following a pathologic femoral fracture secondary to breast metastasis. However, this patient had weakness from multiple lumbar fractures and delay in spinal MRI permitted the development of worsening weakness and cauda equina compression. Principally, orthopedic workup of lower extremity weakness in the setting of a pathologic femoral fracture should include a spinal evaluation, especially if bilateral weakness is present. Additionally described are multiple, currently recommended treatment modalities for prophylaxis or the correction of pathologic fractures of the femur and spine.

## Case presentation

**Figure 1:**
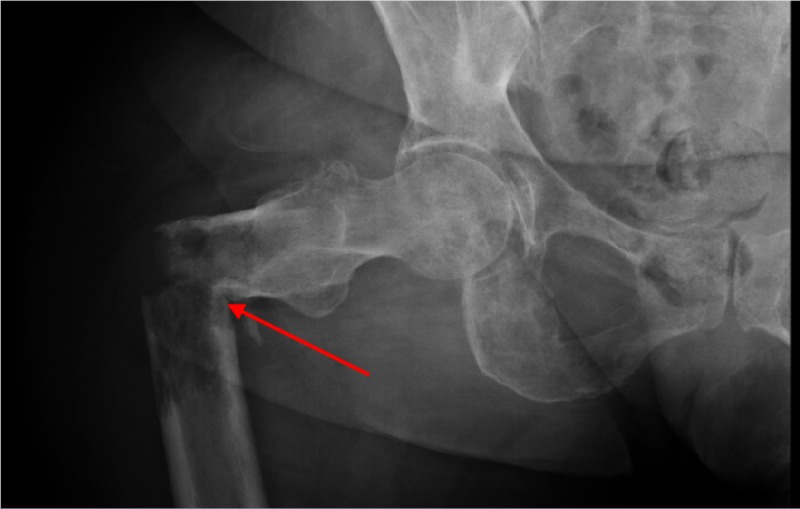
Pathologic fracture of the right proximal femur Acute, transverse, displaced, and angulated right femoral fracture (arrow), with multiple, ill-defined lytic lesions to the right pelvis

**Figure 2:**
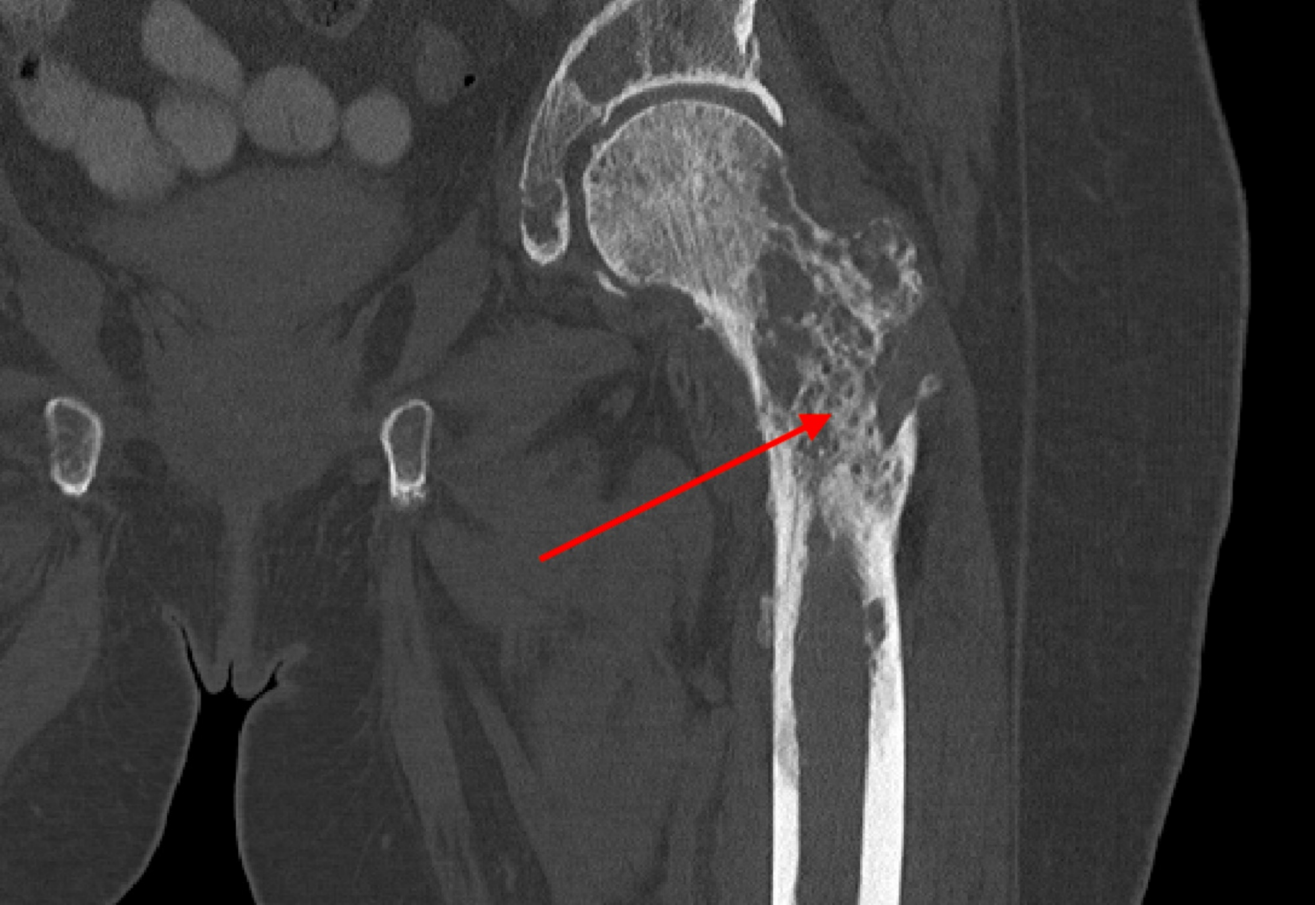
Bony metastasis of the left femur Diffuse bone demineralization, with numerous lytic lesions, noted throughout proximal left femur (arrow). Scattered, metastatic foci likely placing the patient at a higher risk for pathologic fracture.

A 62-year-old woman with no past medical history presented at the behest of her children following an audible “snap” in her right leg while walking at home. Physical examination of the lower extremities was remarkable for bilateral weakness and markedly decreased active range of motion on the right. Imaging revealed an acute, transverse, displaced, and angulated right femoral fracture, with multiple ill-defined lytic lesions to the pelvis and left proximal femur (Figure 1, Figure 2).

**Figure 3:**
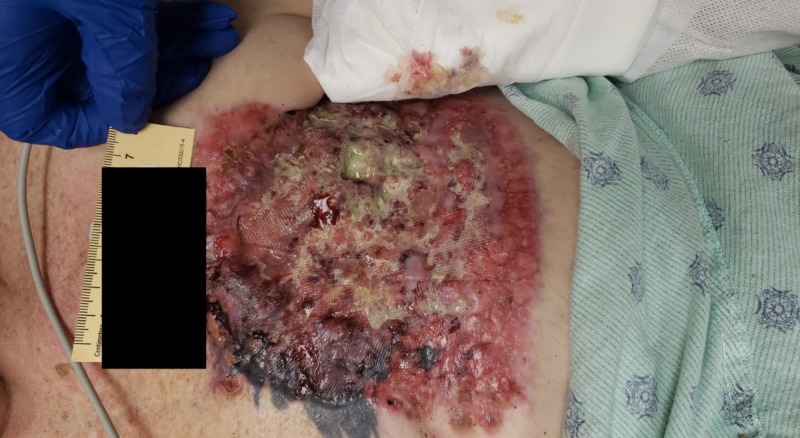
Left chest wall mass 12.5 cm x 16.5 cm x 1.0 cm necrotic, fungating left chest wall wound suggestive of metastatic breast disease

There was no identifiable fracture on the left side. Further examination exposed a necrotic, fungating left chest wall mass (Figure 3), with left axillary lymphadenopathy suggestive of metastatic breast disease.

**Figure 4:**
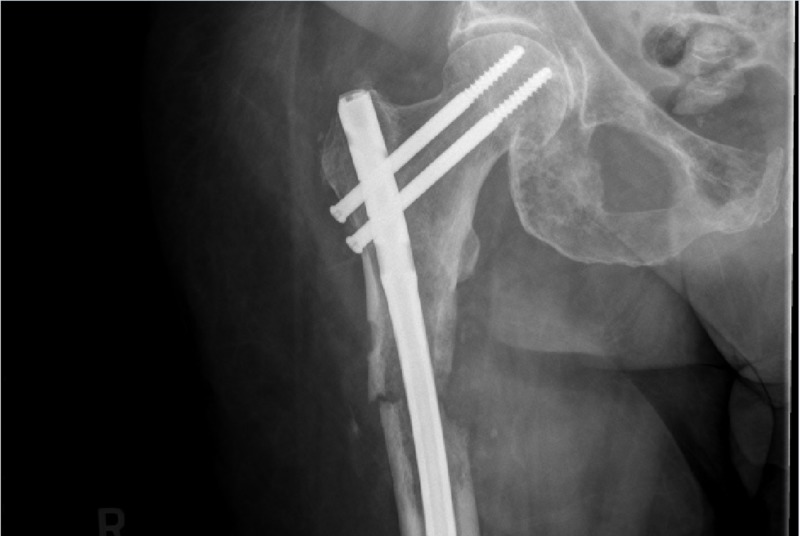
Postoperative right femur Anterograde intramedullary rod with proximal and distal (not seen) interlocking screws stabilizing the fracture site. Anatomic alignment.

The following day, the patient underwent right femoral IMN fixation using a 12 mm x 420 mm titanium cannulated nail. Thereafter, she was neurovascularly intact throughout her right lower extremity and postoperative films showed anatomic alignment (Figure 4).

**Figure 5:**
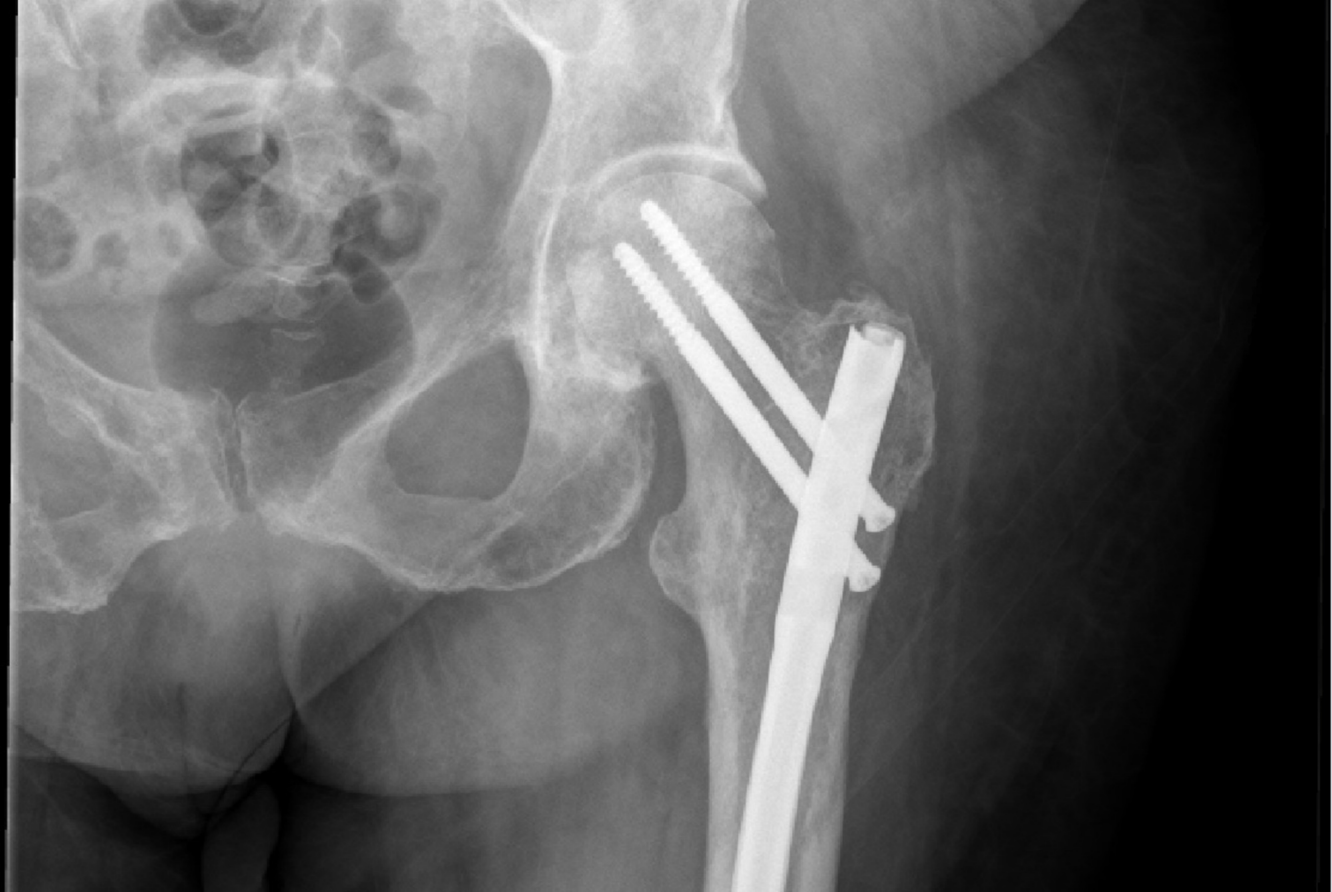
Postoperative left femur Intramedullary rod in the left femur extending from the greater trochanter to the distal metaphysis with proximal interlocking screws and a single distal interlocking screw (not seen). Alignment appears anatomic. No fractures are identified.

Then, two days later, with a Mirels’ score of 8, she underwent prophylactic left femoral IMN fixation. Postoperative films showed anatomic alignment (Figure 5). Following the procedure, she was neurovascularly intact throughout her left lower extremity. On postoperative Day 3 from the left IMN fixation, she was discharged from the orthopedic service.

**Figure 6:**
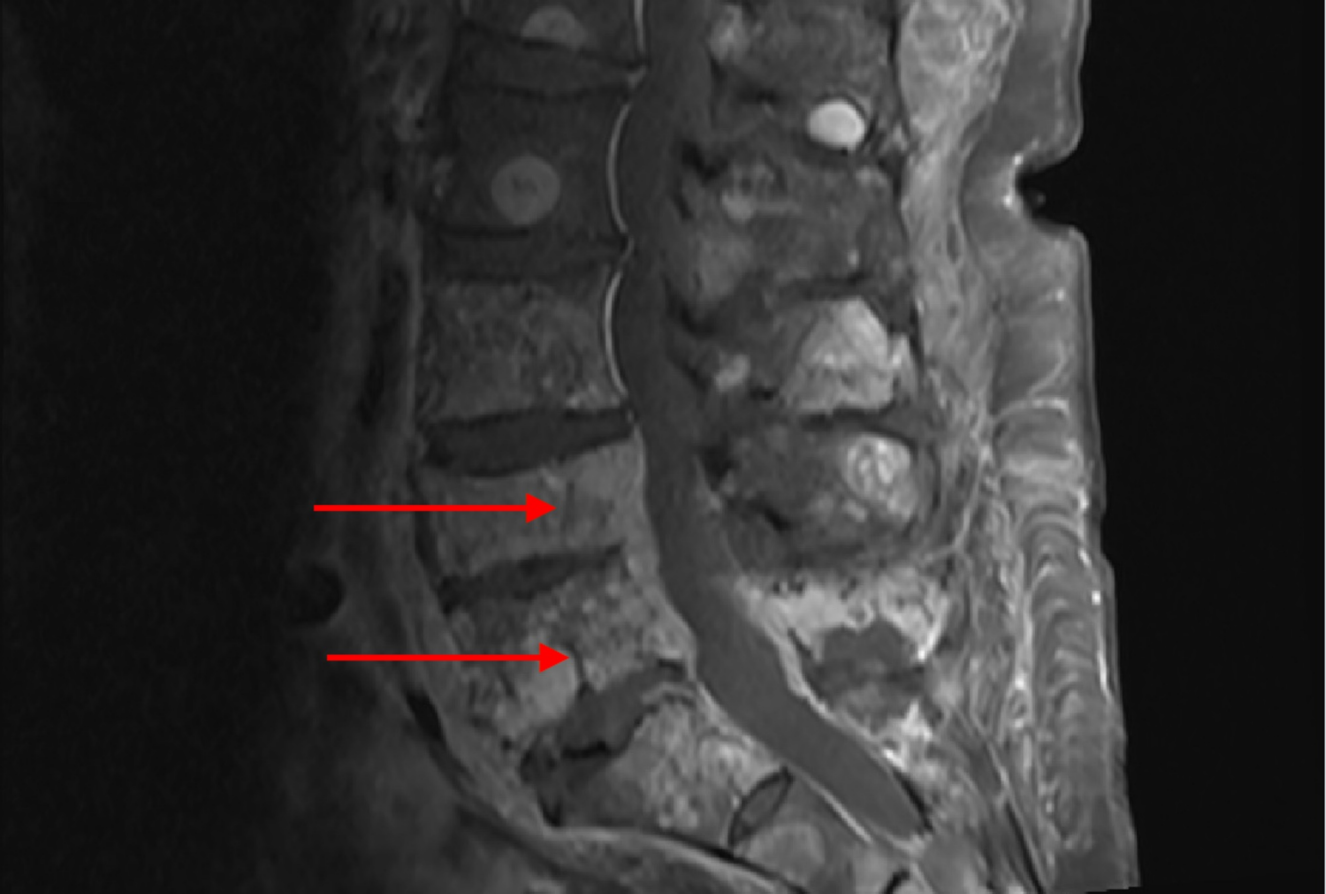
Vertebral compression fractures Severe extensive metastatic disease with pathologic compression fractures of L4 and L5 (arrows) and extensive epidural tumor invasion at L4 and L5 with foraminal neuronal encroachment.

On postoperative Day 5, the patient presented to the rehabilitation unit with worsening bilateral lower extremity weakness and left foot drop. For the next seven days, she showed no improvement, and on the eighth day, she developed urinary frequency and incontinence. Subsequent lumbar MR imaging revealed pathologic compression fractures of L4 and L5, with epidural tumor invasion and neural foraminal encroachment (Figure 6). She was thereafter initiated on a palliative radiotherapy regimen and discharged without any further orthopedic or neurosurgical intervention.

## Discussion

IMN fixation or hip arthroplasty (HA) are two treatment options for pathologic femur fractures that have proven results in providing pain relief and improved functional mobility. In their prospective study, Picciolo et al. demonstrated the efficacy of IMN fixation for pathologic, proximal femoral diaphyseal fractures through subjective pain relief and general postoperative improvement up to six to 10 months [2]. Additionally, research by Guzik et al. identified 122 patients with proximal femoral metastasis who underwent standard or modular endoprosthetic replacement of the proximal femur. Their data highlight a moderate reduction in visual analog scale (VAS) pain scores and improved quality of life at 27 months following surgery, suggesting HA as a viable treatment option [3]. Therefore, there is no doubt that surgical correction is an evidenced-based option for the treatment of pathologic fractures of the proximal femur.

Similarly, IMN fixation or HA can treat impending fractures. Prophylactic surgical fixation of the proximal femur is commonly offered to patients with metastatic disease to mitigate the risk of an impending pathologic fracture. However, as was highlighted in research by McLynn et al., the perioperative complication profile has received little attention. In their retrospective study of 332 cases of prophylactic femoral fixation and 288 cases of pathologic fracture fixation, they report that when controlling for disseminated cancer, the odds of experiencing an adverse event (major or minor), death, or prolonged hospital stay were no greater in prophylactic fixation than in fracture fixation for up to 30 days [4]. Recent data further suggest that patients who undergo prophylactic stabilization have a lower risk of major complications within 30 days postoperatively, and shorter hospital stays when compared to patients who underwent post-fracture stabilization [5]. Despite positive short-term outcomes for both prophylactic and corrective fixation, however, nonunion and/or tumor progression may lead to long-term hardware failure. Additionally, implant fatigue can occur as a result of repetitive radiotherapy. In a study by Johnson et al., 26 patients were noted to undergo conversion hip arthroplasty for the salvage of failed fixation of a pathologic femur fracture, with a greater number of failed patients having undergone IMN fixation (n=18) [6]. Their data report conversion HA at a mean 13 months after initial fixation, most commonly due to disease progression (n=12), hardware failure (n=8), and nonunion (n=6) [6]. Therefore, in the era of prolonged cancer survival, endoprostheses may serve as a reasonable alternative to prophylactic IMN fixation if the patient portends a relatively favorable prognosis.

Our patient was a 62-year-old female who underwent right open-reduction and IMN fixation of a pathologic fracture and prophylactic IMN fixation of the left femur. Given her age and lack of comorbid disease aside from metastatic breast cancer, the initial orthopedic management was likely appropriate, though incomplete. The original films identified heterogeneous, ill-defined lytic lesions of the pelvis. Therefore, the question is whether this patient should have had a more thorough oncologic workup or staging prior to prophylactic surgery on the left, which may have made her a better candidate for endoprostheses. In a study conducted by Wedin and Bauer in 2005, 142 patients were treated surgically for proximal femoral metastatic lesions and, not surprisingly, those treated with IMN fixation had a higher rate of failure (16.2%) when compared to endoprostheses (13.6%) [7]. This data, in conjunction with the anticipated failure rate at 13 months in a similar age-matched cohort as described above, suggest endoprostheses have a greater long-term benefit than prophylactic cephalomedullary nailing. I would argue that our patient may have had more benefit from an endoprosthesis than IMN fixation.

Second, would the earlier identification of severe lumbar degeneration with cord involvement have warranted a more aggressive orthopedic workup such as kyphoplasty, vertebroplasty, and/or fusion with instrumentation? As was mentioned, a delay in lumbar imaging likely lent itself to worsening lower extremity weakness and neurologic compromise, including foot drop and urinary incontinence. Taken about 2.5 weeks after initial presentation, MR imaging identified pathologic compression fractures of L4 and L5, with epidural tumor invasion and neural foraminal encroachment. Despite its insidious time course, the premature introduction of rehabilitation without the identification of spinal pathology may have exacerbated the patient’s symptoms. Additionally, the development of impaired left-sided dorsiflexion with urinary incontinence would likely have been prevented with a more aggressive spinal workup. A review of the current literature on the orthopedic management of malignant vertebral compression fractures (MCVF) suggests surgical intervention, such as kyphoplasty, may be of benefit. In a retrospective study of 48 patients with MVCF treated with kyphoplasty, significant improvements in all outcome measurements were observed postoperatively up to two years following treatment [8]. Additionally, minimally invasive stabilization and circumferential spinal cord decompression exemplified postoperative efficacy in a case report of a patient with osteolytic destruction of the vertebral column from metastatic, epidural spinal cord compression [9]. Therefore, our patient certainly may have benefitted from earlier recognition of her lumbar fractures, which, with proper orthopedic intervention, such as cord decompression or kyphoplasty, may have prevented subsequent neurologic dysfunction. This course of action would likely have decreased her hospital stay, reduced rehabilitation costs, and led to more prompt adjunct radiotherapy.

## Conclusions

Pathologic fractures most commonly occur in the proximal femur, for which there are well-described, orthopedic surgical treatments. In some patients, contralateral weakness suggests an additional fracture. However, bilateral weakness may also indicate spinal involvement, and prompt recognition and/or correction is essential to preserve neurologic function. In this case, lumbar compression fractures were missed, likely permitting neurologic compromise. This case not only emphasizes the importance of a thorough musculoskeletal workup for lower extremity weakness but explores multiple current treatment options for pathologic fractures of the femur and spine and suggests that within the context of a favorable prognosis, endoprostheses may prove advantageous to IMN fixation of the femur.
